# Cystinosis: a review

**DOI:** 10.1186/s13023-016-0426-y

**Published:** 2016-04-22

**Authors:** Mohamed A. Elmonem, Koenraad R. Veys, Neveen A. Soliman, Maria van Dyck, Lambertus P. van den Heuvel, Elena Levtchenko

**Affiliations:** Department of Pediatric Nephrology & Growth and Regeneration, University Hospitals Leuven & KU Leuven, UZ Herestraat 49–3000, Leuven, Belgium; Department of Clinical and Chemical Pathology, Faculty of Medicine, Cairo University, Cairo, Egypt; Department of Pediatrics, Center of Pediatric Nephrology and Transplantation (CPNT), Faculty of Medicine, Cairo University, Cairo, Egypt; EGORD, Egyptian group of orphan renal diseases, Cairo, Egypt; Department of Pediatric Nephrology, Radboud University Medical Center, Nijmegen, The Netherlands

## Abstract

Cystinosis is the most common hereditary cause of renal Fanconi syndrome in children. It is an autosomal recessive lysosomal storage disorder caused by mutations in the *CTNS* gene encoding for the carrier protein cystinosin, transporting cystine out of the lysosomal compartment. Defective cystinosin function leads to intra-lysosomal cystine accumulation in all body cells and organs. The kidneys are initially affected during the first year of life through proximal tubular damage followed by progressive glomerular damage and end stage renal failure during mid-childhood if not treated. Other affected organs include eyes, thyroid, pancreas, gonads, muscles and CNS. Leucocyte cystine assay is the cornerstone for both diagnosis and therapeutic monitoring of the disease. Several lines of treatment are available for cystinosis including the cystine depleting agent cysteamine, renal replacement therapy, hormonal therapy and others; however, no curative treatment is yet available. In the current review we will discuss the most important clinical features of the disease, advantages and disadvantages of the current diagnostic and therapeutic options and the main topics of future research in cystinosis.

## Background

Cystinosis was first described in literature in 1903 by the Swiss biochemist Emil Abderhalden (1877–1950) as the familial cystine accumulation disease [1]. Abderhalden referred to a child initially encountered by Eduard Kaufmann, Basel, Switzerland (1860–1931). This patient died at the age of 21 months with massive cystine accumulation in multiple organs that were discovered at the postmortem examination. The Dutch pathologist George Lignac (1891–1954) was the first to provide a clear systematic description of the disease in 1924, and the first to associate cystinosis with its major clinical manifestations such as rickets, renal disease and growth retardation [2]. This is why cystinosis was initially termed as the Abderhalden-Kaufmann-Lignac syndrome. Guido Fanconi (1892–1979), the Swiss pediatrician, also substantially contributed to the understanding of cystinosis by explaining the urinary substance losing nature of the disease [3]. Hence, cystinosis was also recognized in the literature as the Lignac-Fanconi syndrome.

The currently used term “cystinosis” is a modification from the German term “Cystindiathese” or “hereditary cystine disease” which was initially used by Emil Abderhalden to describe the disease in 1903 and was modified in the English literature to “cystine disease” then “cystinosis”. Cystinosis (ORPHA213) is a rare autosomal recessive lysosomal storage disorder in which the amino acid cystine accumulates in the lysosomes of cells [4]. Cystinosis is one of the few rare diseases having a specific treatment. The aminothiol cysteamine, used for the treatment of cystinosis for over 20 years now [5], can deplete the intralysosomal cystine through the reduction of cystine, and the formation of cysteine and a cysteamine-cysteine mixed disulfide which exits the lysosome via the cationic amino acid transporter PQLC2, thus bypassing the original genetic and biochemical defects of the disease [6, 7].

Cystinosis is a systemic disease and cystine crystals, the pathologic landmark, accumulate in all body cells and tissues. Although cystinosis is a monogenic disease, it has three major clinical presentations depending on the severity of mutations affecting the *CTNS* gene: the infantile nephropathic form (MIM: 219800, ORPHA411629), the juvenile nephropathic form (MIM: 219900, ORPHA411634) and the ocular non-nephropathic form (MIM: 219750, ORPHA411641) [8].

In the current review, we describe the clinical spectrum of the disease, the diagnostic and management protocols and the anticipated advances in the near future.

### Epidemiology

Nationwide birth prevalence data concerning cystinosis are only reported in few populations. The overall incidence rates reported in France [9], Australia [10], Germany [11], Denmark [12] and Sweden [13] were 1:167,000, 1: 192,000, 1: 179,000, 1:115,000 and 1:260,000 live births, respectively. Higher incidence rate is observed in selected populations with detected founder mutations as in the province of Brittany, France (1:26,000 live births) [14] or in the Saguenay-Lac-St-Jean, Quebec, Canada (1:62,500 live births) [15]. The highest birth frequency rate ever reported was in the Pakistani ethnic group living in the West Midlands, UK (1: 3,600) [16]. Since cystinosis is an autosomal recessive disease, its incidence is expected to be affected by the extent of consanguinity in the community. Accurate statistical data about the incidence of cystinosis in regions with high consanguinity such as Middle East and North Africa are still lacking; however, cystinosis was fairly commonly detected among a large cohort of different lysosomal storage disorders diagnosed over a six year period in Egypt (29/211 patients (13.7 %)) [17].

### Etiology

Cystinosis is caused by bi-allelic mutations in the *CTNS* gene (17p13.2) encoding cystinosin, which is a lysosomal cystine-proton co-transporter. Consequently, cystine accumulates in the lysosomes of affected cells and forms crystals in low lysosomal pH [4].

**Fig. 1:**
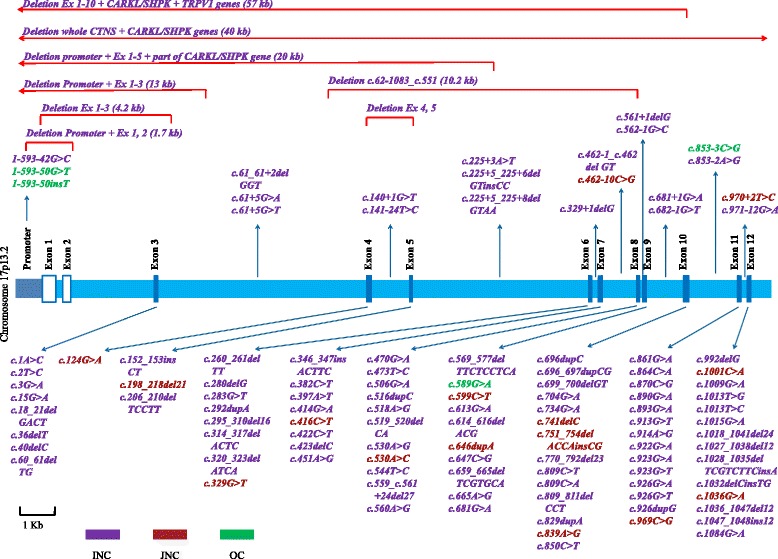
Schematic representation of the *CTNS* gene and all reported mutations in cystinosis patients. Exonic mutations are displayed in the lower half of the figure, while promoter and intronic mutations and large deletions are displayed in the upper half. INC: infantile nephropathic cystinosis, JNC: juvenile nephropathic cystinosis, OC: ocular cystinosis

So far, over 100 pathogenic mutations have been reported in the literature (Fig. 1). The most commonly detected pathogenic mutation is the 57-kb deletion present in almost 50 % of *CTNS* mutant alleles of patients of North European and North American origin [18, 19]; however, outside this geographical distribution, the mutation is almost completely absent, especially in the Middle East [20]. Severe or truncating mutations on both alleles are usually associated with the infantile severe form of the disease, while juvenile and ocular forms of cystinosis are usually associated with at least one mild mutation. Genotypic-phenotypic correlations for the discovered *CTNS* mutations are summarized in Fig. 1.

### Clinical description and complications

#### Renal manifestations

Three clinical forms of cystinosis can be distinguished, depending on the age of presentation and the degree of renal disease severity.

The infantile nephropathic form is the most frequent (95 %) and the most severe type of cystinosis. The renal phenotype consists of renal Fanconi syndrome, and a consecutively progressive loss of glomerular function leading to end-stage renal failure [4, 21, 22]. Asymptomatic aminoaciduria is the first manifestation of renal Fanconi syndrome in humans [23, 24] with urinary losses of amino acids being 6 to 16 times increased compared to the normal amount [25]. The earlier loss of expression of apical proximal tubular receptors megalin/cubilin and SGLT-2, and NaPi-IIa transporters preceding cell atrophy in a mouse model of cystinosis, provides an explanation for the early proteinuria, glucosuria, and phosphaturia [26, 27].

By the age of 6 to 12 months, selective proximal tubular dysfunction develops into the full-blown renal Fanconi syndrome, characterized by excessive urinary loss of amino acids, sodium, potassium, bicarbonate, magnesium, carnitine, calcium, phosphate, glucose and low molecular (LMW) to intermediate molecular weight (IMW) proteins [23, 25]. Infants present with failure to thrive, polyuria, polydipsia, episodes of severe dehydration and electrolyte imbalance, vomiting, constipation and sometimes vitamin D resistant rickets. Laboratory findings may include hypokalemia, metabolic acidosis, hypophosphatemia, hypocalcemia, low carnitine levels and less frequently hyponatremia. However, metabolic alkalosis in case of a Bartter-like presentation has also been reported [28, 29].

**Fig. 2:**
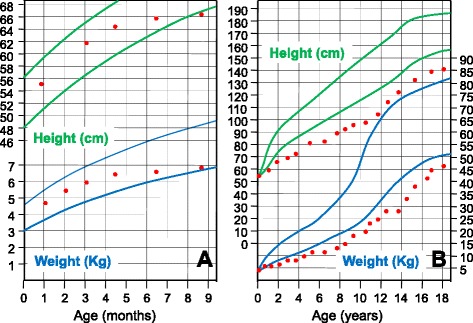
Typical growth charts in 2 cystinosis patients: **a**- Normal growth pattern at birth, followed by decreased growth velocity after six months. **b**- Progressive decrease in growth velocity in a patient who started cysteamine therapy after 2 years of age and was not treated with GH. Green and blue lines represent the 3rd and the 97th percentiles for Height and weight, respectively. (Adapted with permission from Besouw and Levtchenko, 2010) [27]

**Fig. 3:**
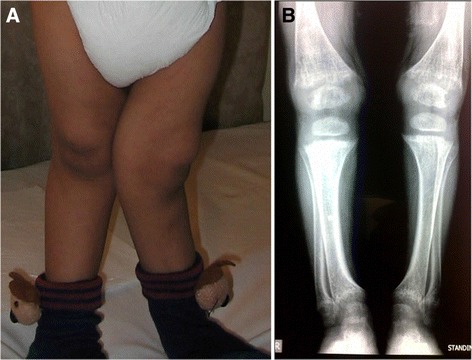
Rickets in cystinosis. **a**- A cystinosis child with evident rachitic bone deformities. **b**- Active rachitic bone disease in X-Rays

At birth, patients show a normal birth length and weight parameters. By the age of 6 to 12 months, height drops to the third percentile, and further growth is restricted to less than 60 % of the normal range [8, 30, 31]. Figure 2 represents the typical growth pattern of cystinosis patients if specific treatment is not started in the first year of life. Calciuria and phosphaturia, in combination with phosphate, calcium, vitamin D and alkalinizing agent supplementation can cause medullary nephrocalcinosis and nephrolithiasis in a subset of patients [32]. Phosphaturia, increased urinary losses of vitamin D binding protein and decreased renal vitamin D conversion due to decreased activity of alpha-1 hydroxylase in renal proximal tubules, can lead to vitamin D resistant hypophosphatemic rickets in children (Fig. 3) and osteomalacia in adults [33]. Proteinuria is variable, and consists initially of low molecular weight proteins (beta-2-microglobulin, alfa-1-microglobulin, retinol-binding protein) and intermediate weight proteins (albumin, transferrin, vitamin D binding protein). Glomerular proteinuria is present starting from early ages and is characterized by excessive urinary losses of albumin and HMW proteins, and may occur up to nephrotic range [34]. Generally, serum creatinine levels remain within normal limits until the age of 5 years and only rarely exceed 1 mg/dl below this age [8, 35]. If left untreated or if treatment started late or even if the patient was not compliant to treatment, end stage renal disease (ESRD) develops by the end of the first decade of life [36, 37]. Due to the severe polyuria, episodes of dehydration can be detrimental and usually accelerate the onset of ESRD at young age.

A small group of cystinosis patients (5 %) is diagnosed during late childhood or adolescence with the juvenile (late onset) form of cystinosis [38]. Patients present with a variable spectrum of features, ranging from isolated asymptomatic proteinuria, a mild renal Fanconi syndrome, to an overt nephrotic syndrome and usually they do not develop remarkable growth retardation. Generally, there is a slower progression rate to ESRD and extra-renal complications. In small series, four out of 14 patients with juvenile cystinosis developed ESRD at 12, 21, 27 and 28 years of age [38].

The adult, non-nephropathic ocular form of cystinosis is characterized only by photophobia due to corneal cystine accumulation, and rarely presents before adulthood [39]. The kidneys and other organs are spared from symptoms. In one family the co-existence of ocular and late-onset forms of cystinosis has been reported, implying the need of regular renal function controls in patients with ocular cystinosis [38].

### Extra-renal manifestations

Being a systemic lysosomal storage disorder cystinosis manifests in almost all tissues and organs, and, although most systemic features are manifested relatively late during the course of the disease, the pathological processes behind these manifestations, especially cystine accumulation, usually start early. Nearly all nephropathic cystinosis patients who did not receive early cystine-depleting therapy or those who are not compliant, will develop major extra-renal symptoms including retinal, endocrinological and neuromuscular complications by the age of 30 years [40].

**Fig. 4:**
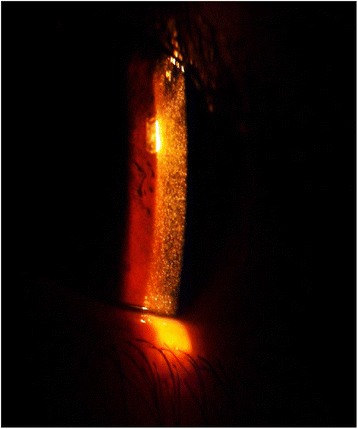
Corneal cystine crystals. Slit lamp examination of corneal cystine deposits (courtesy of Prof. Dr. Akmal Rizk, Dr. Mohamed Gamal and Prof. Dr. Neveen Soliman)

Corneal cystine accumulation with crystal formation is the first extra-renal finding affecting all cystinosis patients [8]. It leads to photophobia and blepharospasm usually between mid-childhood to early adolescence. [8, 41]. At birth, corneal cystine crystals are not detectable. They can only be observed from the age of 12 months through a slit lamp examination by an experienced ophthalmologist and are always present by the age of 18 months (Fig. 4). While superficial punctate and filamentary keratopathy is frequently seen in adolescent and adult patients, band keratopathy, peripheral corneal neovascularization and posterior synechiae associated with iris thickening are mostly found in older patients [42]. Depigmentation of the peripheral retina with pigment epithelial mottling is a commonly encountered posterior segment complication [43]. It presents mainly from the second decade of life, but has already been observed as early as at 6 months of age. In 10–15 % of patients, retinopathy leads to retinal blindness [44].

Progressive cystine accumulation and crystal formation in thyroid follicular cells causes fibrosis and atrophy leading to primary hypothyroidism [45, 46], manifesting in the majority of cystinosis patients (50–70 %) from the second decade of life [30]. Earlier thyroid changes affecting thyroglobulin synthesis and iodo-thyroglobulin processing might be responsible for subclinical hypothyroidism with TSH elevation and normal T3 and T4 plasma concentrations, as it has been shown in a mouse model of cystinosis [47].

Endocrine and exocrine pancreatic insufficiency have been also reported in cystinotic patients, usually after renal allograft transplantation [48, 49]. Fifty percent of infantile cystinosis patients by the age of 18 develop slow progressive loss of insulin secretion and C-peptide production leading to glucose intolerance and diabetes mellitus [50]. Hepatomegaly and/or splenomegaly are present in about one third of patients by the age of 15 years, however, liver function usually remains unaffected [48].

In male cystinosis patients, primary hypogonadism is a frequent finding (70 %) [51–53]. Recently, Besouw et al. have shown that although azoospermia was present in all studied male cystinosis patients under cysteamine therapy, spermatogenesis was documented on a testicular biopsy specimen in one renal transplant patient [54]. In females, although delayed puberty is sometimes observed, normal pubertal development is also possible and in contrast to males, cystinotic females are usually fertile [55].

Central nervous system involvement is evident in a subgroup of cystinotic patients and becomes more frequent with advancing age. Neurological findings include hypotonia, tremor, speech delay, gross and fine motor impairment, idiopathic intracranial hypertension, neurocognitive dysfunction, behavioral problems and encephalopathy [56–61]. Despite normal IQ scores, cystinosis patients have significantly poorer performance in visual spatial and visual memory skills than normal individuals and, interestingly, their highest scores are in the area of auditory short-term memory, which could be a compensatory mechanism for their poor visual memory [58, 62]. A recent study in cystinosis patients aged 3–7 years, using the MRI based technique, diffusion tensor imaging (DTI) detected the early selective white matter microstructural changes in the form of bilaterally decreased fractional anisotrophy and increased mean diffusivity in the inferior and superior parietal lobules in children with cystinosis corresponding to the areas of the dorsal and ventral visual pathways [63], thus giving the pathological explanation for the early onset of poor visual spatial and visual memory skills. Other common pathological findings usually observed at older age include cerebral cortical atrophy, non-absorptive hydrocephalus, demyelination, and vacuolar, necrotic and spongiform changes [57, 58].

A distal vacuolar myopathy presenting as progressive distal muscle wasting and weakness, has been observed in about 24 % of renal transplant cystinosis patients [64, 65]. Myopathy generally affects patients from their second decade of life. Myopathy changes on EMG can be present in asymptomatic patients, suggesting that clinically overt muscle weakness might be a late sign of cystinotic myopathy [60]. In post-transplant cystinotic patients who did not receive long-term cystine-depleting therapy, cystinotic myopathy may cause an extraparenchymal pattern of restrictive lung disease [66]. Swallowing dysfunction occurs in more than half of patients with myopathy and its severity also positively correlates with the number of years without cysteamine therapy [67]. As a result, aspiration pneumonia constitutes a severe and potentially lethal complication.

Other observed features related to skin, hair and salivary glands have also been reported such as congenital hypopigmentation, premature skin ageing, impaired sweating and salivation and progressive coarse facial features due to subcutaneous cystine infiltration [68]. According to our experience, not only European patients, but also some patients from other ethnic backgrounds can present with characteristic blond hair and white skin. Recently, cystinosin was implicated in the regulation of melanin synthesis as *CTNS* silencing in a melanoma cell model led to over 50 % reduction in pigment production [69].

### Differential diagnosis

Although cystinosis is the most common identifiable cause of the inherited renal Fanconi syndrome in children, other metabolic diseases (tyrosinemia, galactosemia, glycogen storage diseases), Wilson’s disease, Dent’s disease and Lowe’s syndrome should also be considered in the differential diagnosis of the renal Fanconi syndrome. Some cystinosis patients had atypical presentations and were initially diagnosed as Bartter’s syndrome or nephrogenic diabetes insipidus [28, 29]. Most frequent genetic and acquired conditions for the differential diagnosis of cystinosis are summarized in Table 1.

**Table 1:** Differential diagnosis of cystinosis according to the most common presenting manifestations

Presenting manifestations	Diseases	MIM	Gene	Protein	Other characteristic features at presentation
Proximal renal tubular acidosis	Tyrosinemia type I	276700	*FAH*	Fumarylacetoacetase	Hepatomegaly, mental retardation
	Galactosemia	230400	*GALT*	Galactose-1-phosphate uridylyltransferase	Lethargy, jaundice, bleeding disorders, cataract, intellectual disability
	Hereditary fructose intolerance	229600	*ALDOB*	Aldolase B	Seizures, irritability, poor feeding, lethargy, liver disease
	Wilson disease	277900	*ATP7B*	Copper transporting P-type ATPase	Liver disease, neuropsychiatric manifestations, Kayser-Fleischer ring in the cornea
	Lowe syndrome	309000	*OCRL*	Phosphatidylinositol 4,5-diphosphate 5-phosphatase	Congenital cataract, glaucoma, intellectual disability, hypotonia, seizures, behavioral problems
	Dent’s disease	300009	*CLCN5*	Chloride Channel Protein number 5	Low molecular weight proteinuria, hypercalciuria, nephrolithiasis, nephrocalcinosis, progressive renal failure
	Mitochondrial disorders:				
	- Leigh syndrome	256000	*COX10*	Cytochrome C oxidase assembly protein	Encephalopathy, myopathy, respiratory istress, deterioration of cognitive function
	- Gracile syndrome	603358	*BCS1L*	S. cerevisiae bcs1 protein homolog	Severe lactic acidosis, hypoglycemia, cholestasis, iron overload
	- HUPRA syndrome	613845	*SARS2*	Seryl-t-RNA synthetase	Hyperuricemia, pulmonary hypertension, renal failure, alkalosis
	- Mitochondrial DNA depletion syndrome 8	612075	*RRM2B*	Ribonucleotide reductase small subunit 2 like	Neonatal hypotonia, lactic acidosis, neurologic deterioration
	- Mitochondrial DNA depletion syndrome 13	615471	*FBXL4*	Leucine rich repeat protein 4	Hypotonia, lactic acidois, microcephaly, congenital cataract
	Heavy metal toxicity: Lead, cadmium	----------	-----------	---------------------------------------	Anemia, abdominal pain, encephalopathy, osteomalacia, neurological manifestations
Hypophosphatemic Rickets	Hypophosphatemic nephrolithiasis/osteoporosis I	612286	*SLC34A1*	Sodium-phosphate cotransporter, member 1	Nephrolithiasis, osteoporosis, multiple fractures
	Hypophosphatemic nephrolithiasis/osteoporosis II	612287	*SLC9A3R1*	Sodium/hydrogen exchanger regulatory factor 1	Nephrolithiasis, osteoporosis, hypocalcemia, hypoparathyroidism
	Autosomal dominant hypophosphatemic rickets	193100	*FGF23*	Fibroblast growth factor 23	Fatigue, bony pains, bone deformities
	Autosomal recessive hypophosphatemic rickets	241520	*DMP1*	Dentin matrix acidic phosphoprotein 1	Retarded skeletal growth, abnormal mineralization
	Hereditary hypophosphatemic rickets with hypercalciuria	241530	*SLC34A3*	Sodium-phosphate cotransporter, member 3	Elevated serum 1,25-dihydroxy vitamin D levels, hypercalciuria, osteomalacia, nephrolithiasis, nephrocalcinosis
	Vitamin D dependent rickets type I	264700	*CYP27B1*	25-hydroxyvitamin D3-1-alpha-hydroxylase	Hypotonia, muscle weakness, seizures
	Vitamin D dependent rickets type II	277440	*VDR*	vitamin D receptor	Alopecia, hypocalcemia, secondary hyperparathyroidism, osteomalacia, osteitis fibrosa cystica
Stunted growth	Cystic fibrosis	219700	*CFTR*	Cystic fibrosis transmembrane conductance regulator protein	Frequent chest infections, pancreatic insufficiency
	Chronic malnutrition	----------	-----------	---------------------------------------	Fatigue, anemia, poor cognitive function, behavioral changes, history of poor socioeconomic standard
	Hormonal causes				
	- Hypothyroidism	----------	-----------	---------------------------------------	Lethargy, fatigue, dry skin, cold intolerance, constipation, mental subnormality
	- GH deficiency	----------	-----------	---------------------------------------	Short stature with general good health, normal intelligence
	Familial	----------	-----------	---------------------------------------	Family history

Cystinosis is also responsible for some cases of childhood renal failure, and should be considered in every young patient presenting with renal failure of unknown origin [37].

### Diagnostic methods

Due to the availability of specific cysteamine therapy, early diagnosis and management of cystinosis have a great impact on the clinical outcome of patients. There are three main diagnostic modalities for cystinosis. The current gold standard is the detection of elevated cystine levels in white blood cells (WBCs), being extremely sensitive and precise for the disease. Molecular testing of the relatively small *CTNS* gene (12 exons but only 10 are coding) is also a well- established technique revealing 95 % of disease causing mutations. The third clinically used confirmatory option is the detection of the characteristic corneal cystine crystals by slit lamp examination [27].

Oshima et al. established a highly sensitive and specific method for cystine measurement in WBCs in 1974 [70]. Their assay was based on the selective binding between cystine in the WBC sample and cystine binding protein (CBP) isolated from *Escherichia coli* in the presence of a competitor external [^14^C] cystine, with the resultant bound radioactivity being inversely proportional to cystine concentration in the unknown sample. Although still used in few laboratories, this method is widely replaced now by high performance liquid chromatography (HPLC) or liquid chromatography-tandem mass spectrometry (LC-MS/MS) methods [71, 72]. Newly diagnosed cystinotic patients have WBC cystine levels in the range of 3–20 nmol half-cystine/mg protein, while control individuals and heterozygous carriers have levels <0.2 and <1.0 nmol half-cystine/mg protein, respectively. The main source of the assay variability lies in the method applied for WBCs separation, and whether it is a mixed leucocyte or polymorphonuclear leucocyte population, thus, the type of cells and the separation method should be highly standardized for each laboratory. Nevertheless, the instrumentations and techniques involved, so far, in cystine measurement are sophisticated enough to limit its use to few university hospitals and research centers mainly in the developed world. This is further complicated by the sample sensitivity to storage and transportation conditions, therefore so far most developing nations are still lacking the assay [73].

Being a monogenic disease, molecular diagnosis is efficient as a confirmatory tool; however, it is usually more time consuming than cystine measurement. In about 5 % of patients pathogenic mutations are not easily discovered by the usual *CTNS* gene sequencing, being either deeply intronic, in the promoter region or not commonly encountered large deletion or duplication [74].

The visualization of corneal cystine crystals is the main diagnostic method for cystinosis in developing nations. Although reliable and relatively cheap, it needs a considerably experienced ophthalmologist to identify and grade the crystals properly. Cystine crystals also do not appear in the slit lamp examination until the second year of life which delays the start of specific therapy in most patients [75].

Another possible non-invasive evaluator of the cystine crystal load is the reflectance confocal microscopy (RCM). Chiaverini et al. have shown that RCM is able to detect dermal cystine deposition in young patients with cystinosis [76]. Cystine deposits were visualized as bright, round, or oval-shaped dermal particles of variable size. These particles appear to be specific in cystinosis, as no particles were identified in control subjects. The nature of the deposits was further confirmed by electron microscopy showing that the particles corresponded to crystalline cystine within fibroblasts in the reticular dermis [76]. The test is rapid (5 min), painless and tolerable even in the youngest children.

Because of the availability of cystine-depleting therapy, the development of a newborn screening method is very tempting; however, it is not available so far. The evaluation of cystine levels in blood spots would be logical to consider but many technical difficulties stand in the way including the much higher sensitive instrumentation needed to quantify cystine reliably in a minute amount of sample (the current ideal sample for WBC cystine is 5–10 ml of blood), the difficulty to prevent the spontaneous oxidation of cysteine into cystine in stored blood spots and the interference from serum cystine levels which are completely not related to the disease [77]. A recent study has detected the elevation of sedoheptulose in dried blood spots of patients homozygous for the 57-kb deletion mutation by tandem mass spectrometry [78]. This is the result of the simultaneous deletion of the *CTNS* upstream gene (*CARKL/SHPK*) which encodes the enzyme sedoheptulokinase. Nonetheless, cystinotic patients not harboring this mutation, or even heterozygous would have a completely normal blood spot sedoheptulose levels, making the clinical use of this method very limited.

Detection of most *CTNS* mutations is accomplished by sequencing individual exons and the adjacent splice sites, while large deletions and insertions could be detected by other molecular techniques such as allele specific PCR, multiplex ligation-dependent probe amplification (MLPA) or fluorescence in-situ hybridization (FISH) [79–81].

Antenatal diagnosis of cystinosis through the detection of elevated cystine in cells of fetal origin has been available for many years now [82]. Two main sample types are used for this purpose, either chorionic villous biopsy sample (taken at 8–9 weeks of gestation) or cultured amniotic cells (14–16 weeks of gestation). Cystine can be directly quantified in these cells with either HPLC or LC-MS/MS [83]. DNA analysis for detecting mutant alleles is currently the most frequently used antenatal screening method, but to reach a molecular diagnosis in a timely fashion for an informed decision, knowing the pathogenic mutation(s) in a previous sibling is highly favored.

### Management

Optimal symptomatic treatment of the renal Fanconi syndrome and extra-renal complications, combined with cysteamine, the specific cystine-depleting therapy represent the mainstay of cystinosis treatment [84]. Early diagnosis is of vital importance to ensure better control of cystinosis as the early start of specific treatment ensures better growth and delays the onset of ESRD and most of extra renal complications.

### Symptomatic treatment

The supportive, symptomatic treatment of cystinosis aims to (1) maintain an adequate fluid- and electrolyte substitution and safeguard the acid–base balance, (2) provide nutritional support, (3) prevent the development of rickets and (4) ensure adequate substitution of needed hormones.

Due to their polyuria and impaired sweating ability, cystinosis patients should have access to water and toilets at all times, and should avoid excessive exposure to heat and sun in order to maintain proper hydration [27]. Electrolyte substitution is provided through oral solutions of sodium bicarbonate or sodium/potassium citrate. Substitution with sodium or potassium phosphate and 1–25-(OH)_2_ cholecalciferol should be initiated from early childhood to compensate for the phosphate imbalance and to prevent rickets in patients with preserved GFR. Sodium, potassium, bicarbonate and phosphate need to be monitored frequently, and the dose of substitution needs to be adjusted accordingly. If phosphate, 1,25-(OH) _2_ cholecalciferol and bicarbonate are excessively substituted, nephrocalcinosis may occur [85, 86]. There is no consensus on the systematic use of indomethacin in order to enhance sodium reabsorption at the ascending limb of the loop of Henle and the collecting ducts [85]; however, this treatment can be useful to decrease polyuria and reduce electrolyte losses.

Carnitine replacement has been suggested because of low plasma and muscle carnitine levels in cystinosis patients, though clinical improvement with this therapy has not been proven yet [87, 88]. The majority of cystinosis patients experience a progressive failure to thrive for which a high-caloric diet is recommended in association with other lines of treatment [89]. Feeding by nasogastric tube should be considered early, especially in children with anorexia, complaints of anorexia and frequent vomiting, or to facilitate administration of medical treatment.

Because of the multiple endocrinopathies caused by cystinosis, hormone replacement therapy plays an important role in symptomatic care. Careful monitoring of the thyroid, and later pancreatic function during childhood and adolescence, is important. In the absence of poor cystine-depleting therapy and renal insufficiency, growth hormone replacement therapy can be considered to prevent growth retardation despite a normal growth hormone axis [90]; however, the long term implications of growth hormone replacement in patients with cystinosis is unclear. Currently, insufficient data is available on the pathophysiology of the azoospermia observed in male cystinosis patients. Testosterone supplementation is indicated in patients with a primary testicular failure and low plasma testosterone levels [54].

Angiotensin converting enzyme inhibitors (ACE inhibitors) are a well-established treatment to reduce proteinuria of glomerular origin and to slow down the decline of glomerular filtration rate in chronic renal failure. Greco et al. reported over 20 years of follow up in cystinosis patients, during which the use of ACE inhibitors was associated with slower deterioration of renal function [91]; however, because of the risk of hypotension and consequent renal function decline, ACE inhibitors must be used with caution in patients with extracellular volume and sodium depletion [92]. The combined use of ACE inhibitors together with indomethacin should be strictly avoided.

In case of renal failure, renal transplantation is the treatment of choice. As renal disease does not recur in the transplanted kidney, kidney graft cures the ESRD, but has no effect on the multi-systemic complications. Therefore, cystine-depleting therapy has to be taken lifelong. Although cystine crystals have been observed in the renal graft, they are of no pathological nor clinical significance since they arise from the host mononuclear cells [93]. In comparison to other renal diseases, renal graft survival in cystinotic patients has been reported as superior, although this has not been demonstrated in ERA-EDTA registries [94–97].

Since fluid and electrolyte losses generally decrease during renal replacement therapy, nephrectomy of the native kidneys is rarely needed. As other post-transplant patients, all cystinosis patients should be monitored for immunodeficiency and infections related to immunosuppressive agents after renal transplantation.

### Cystine-depleting therapy

The aminothiol cysteamine (beta-mercaptoethylamine) is currently the only target-specific treatment for cystinosis patients. It aims to deplete lysosomal cystine in all body cells and tissues. The most commonly used cysteamine preparation is the immediate-release cysteamine bitartrate (IR-CYS) (Cystagon®, Mylan Pharma, Morgantown, WV, USA and Orphan Europe, Paris, France). The drug has been approved for clinical use in cystinosis in 1994 in the USA, and in 1997 in Europe [98].

Cysteamine improves overall prognosis by delaying the progression to end-stage renal disease by 6 to 10 years, and thus the need for renal transplantation during childhood [8, 30, 85, 99–103]. Cysteamine has also been shown to prevent or postpone the development of some extra-renal complications. It reduces the need for thyroid hormone replacement therapy, depletes the muscle parenchyma of cystine hereby reducing myopathy, delays pulmonary and pancreatic dysfunction and prevents growth failure when initiated in early infancy [30, 99–101]. In a large cohort of adult cystinotic patients, the frequency of diabetes and myopathy decreased from 28 % to 0 % and from 60 % to 0 %, respectively when the duration of cysteamine treatment exceeded 20 years, while the percentage of hypothyroidism decreased from 87 % to 56 % in patients having cysteamine for more than 8 years [100].

**Fig. 5:**
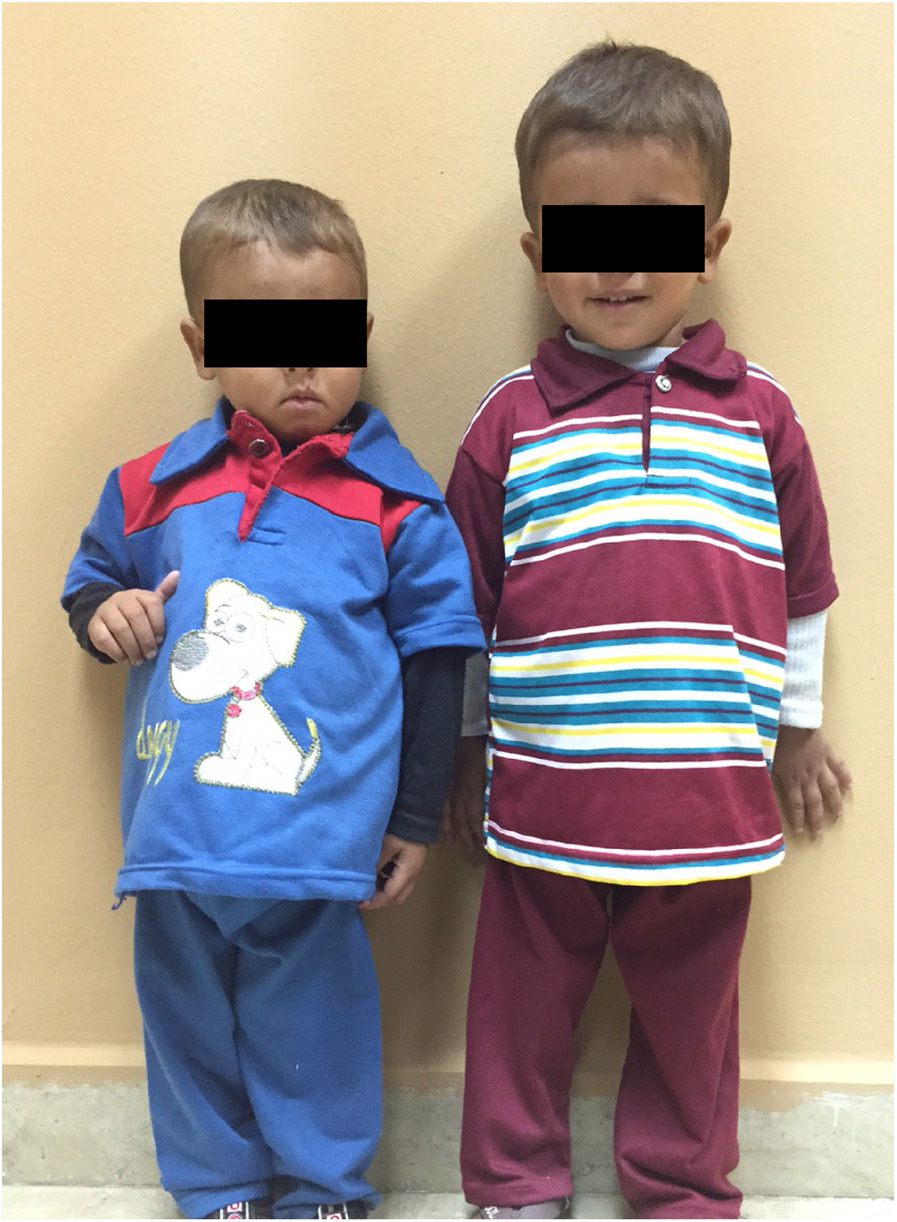
The effect of cysteamine treatment in two siblings with nephropathic cystinosis. The growth in the 30-months old younger sibling (right side, 86 cm, 11.5 kg) who received pre-symptomatic cysteamine therapy at 3 months of age exceeded that of his 56-months old elder brother (left side, 80 cm, 10.5 kg) with later diagnosis and treatment at the age of 20 months (courtesy of Dr. Rasha Helmy and Prof. Dr. Neveen Soliman)

Cysteamine has been shown to prevent growth retardation if initiated in early infancy [31, 97, 104]. However, it is unable to induce a catch-up growth when growth retardation has already set in [90, 91]. Figure 5 demonstrates the importance of early cysteamine therapy to prevent growth retardation. Therefore, cysteamine treatment should be started as soon as possible, and needs to be continued lifelong. In general, the effect of cysteamine in infantile cystinosis is at its best when treatment is initiated in the first year of life, compliance was maintained and leucocyte cystine levels were kept below 1 nmol ½ cystine/mg protein [36]. However, Oral cysteamine has no effect on the renal Fanconi syndrome, male infertility or corneal cystine accumulation [8]. Topical cysteamine therapy can be used to dissolve the corneal cystine crystals, thus treating the photophobia. Cysteamine eye drops are recommended to be used frequently (>10 times per day), but due to acidic formulation are frequently associated with a burning sensation that is especially annoying for children, hampering the compliance. An ophthalmic gel formulation has been recently developed (Cystadrops®, Orphan Europe, Paris, France) and proven to be effective when administered 4 times a day (0.55 %, one drop in each eye per dose) [105]. Ocular symptoms have shown to improve in a couple of weeks, and corneas become clear within few months.

The leucocyte cystine level is today the only available biomarker for monitoring the effectiveness of oral cystine-depleting therapy. The tissue cystine levels at which progressive renal failure and extra-renal complications can be prevented, are unknown. Hence, the 90th percentile of cystine levels in polymorphonuclear cells (<1 nmol ½ cystine per mg protein), seen in heterozygotes, is used as the upper cystine limit. The leucocyte cystine content returns to its initial levels about 6 h after the last administration, therefore the immediate release formulation of cysteamine bitartrate (IR-CYS) has to be taken at a 6-h interval. Greater compliance with oral cysteamine therapy yields greater preservation of renal glomerular function as for every year of excellent cystine depletion, nearly one year of renal function was preserved [106].

Cysteamine is a potent gastric acid-secretagogue and has been used to induce duodenal ulceration in laboratory animals [85]. In children, a fourfold increase in gastric acid production and 50 % increase in serum gastrin levels in comparison to baseline levels, have been reported [107, 108]. Hence, gastrointestinal complaints as nausea, dyspepsia, vomiting and epigastric pain are frequent and cause cysteamine intolerance in approximately 14 % of patients [30, 99, 109]. In our experience, these complaints can be minimized by the low start and gradual increase in cysteamine dosage. Use of proton pump inhibitors has been found effective in the management of gastric acid hypersecretion and ulcerogenicity [110]. In selected patients, a Nissen fundoplication may be of help. A small amount of cysteamine is also metabolized into sulfur-containing compounds (dimethylsulfide, methanethiol), which cause halitosis and a bad sweat odor. For this, oral supplements of riboflavin and chlorophyll tablets are used by some patients [84, 111].

It has been suggested to calculate the cysteamine dose based on body surface area (1.30 g/m^2^/day; maximum of 1.95 g/m^2^/day) instead of body weight (50 mg/kg/day), to avoid overdosing [112]. Recently, some patients treated with high cysteamine doses (>1.95 g/m^2^/day) were reported to have skin striae, bone pain, myalgia, and endothelial proliferative lesions on the elbows showing reactive angioendotheliomatosis on skin biopsy. These adverse events developed in a small proportion of patients, and, while bone and joint pain remained in some patients, cutaneous manifestations resolved after lowering the dose of cysteamine [113].

Other reported adverse effects of cysteamine include hyperthermia, lethargy, neutropenia, seizures and allergic rash [114]. Fortunately, these effects are reversible, and when cysteamine is started at a low dose and increased gradually, these complaints can be prevented [114]. Based on the observation of dose-related (100–150 mg/kg/day) development of a cleft palate, kyphosis, intrauterine growth retardation and intrauterine death with cysteamine treatment in the rat, it is recommended to discontinue cysteamine in women planning pregnancy [115, 116]. The potential risks of cysteamine discontinuation for several months should be carefully balanced against the desire to have children.

Taken together, current cysteamine therapy with its strict dosing regimen and significant adverse effects, imposes a significant burden on cystinosis patients. It has been estimated that only one third of patients are able to adhere to the strict dosing schedule [102]. Poor compliance leads to a less favorable prognosis with progressive renal function deterioration and poor growth [109].

Recently, a new twice-daily delayed-release enteric-coated formula of cysteamine bitartrate (DR-CYS) (Procysbi™, Raptor Pharmaceuticals Inc., Novato, CA, USA) has been approved for clinical use by the US Food and Drug Administration (FDA) and European Medicines Agency (EMA) in 2013 for the treatment of cystinosis. It was developed based on the observation that direct administration of cysteamine in the small intestine resulted in higher plasma concentrations and a higher area under the curve in comparison to administration in the stomach or colon [117]. It has been hypothesized that the greater surface area and the improved absorption rate from the small intestine, and less first pass metabolism, can explain this finding [118]. This new formulation consists of an enteric-coated capsule, containing microspheronized beads. It only needs to be administered twice daily, instead of four times daily. DR-CYS has the potential to improve compliance through its better dosing regimen. Table 2 provides the most important guidelines for the management of cystinosis [84].

**Table 2:** Treatment guidelines for cystinosis

	Medication	Daily dose	Frequency	Remarks
Symptomatic treatment				
Renal Fanconi syndrome				
Polyuria		Free water supply	Day and night	Special attention for sufficient hydration in case of fever, diarrhea and external heat
				Early tube feeding may be needed for water requirements
Malnutrition	high caloric intake	130 % of RDI		Tube feeding can be needed in young infants
Renal salt loosing	sodium citrate or sodium bicarbonate	Oral 2–10 mmol/kg	QID	Between meals
Alkali losses	citrate or bicarbonate as sodium & potassium salts	Oral 5–15 mmol/kg	QID	Normal bicarbonate level (21–24 mmol/l) should be achieved^a^
Potassium losses	potassium citrate or potassium chloride	Oral 2–10 mmol/kg	QID	Potassium level > 3 mmol/l should be achieved^a^
Phosphate losses	sodium or potassium phosphate	Oral 30–60 mg elementary P/kg	QID	Normal age-related phosphate levels should be achieved^a^
				High doses of phosphate supplements can cause or aggravate nephrocalcinosis
Treatment of rickets	calcidiol	Oral 10–25 μg	QD	Follow-up serum calcium concentration to prevent hypercalcemia
	alpha-calcidol or calcitriol	Oral 0.04–0.08 μg/kg		
Copper deficiency	copper supplementation	no data is available in cystinosis		1–10 mg/day depending on age and serum copper levels
				Chlorophyllin tablets that are used to mitigate halitosis contain 4 mg of elemental copper per tablet
Difficult to control electrolyte losses and polyuria	indomethacin	Oral 1–3 mg/kg	BID	Follow-up serum creatinine
				Discontinue in case of dehydration
				Concomitant use with ACE inhibitors is contra-indicated
Carnitine losses	L- carnitine	Oral 20–50 mg/kg	TID	Not proven effect on clinically relevant muscle health
Proteinuria	ACE-inhibitors (enalapril)	Oral 0.10–0.25 mg/kg (for enalapril)	QD	Control serum creatinine and potassium administration at night to avoid hypotension complaints
				Concomitant use with Indomethacin is contra-indicated
Hormonal substitution				
Hypothyroidism	levothyroxin	Oral	QD	Start by 25 % of the recommended dose and increase to full dose in 4 weeks
		<12 years:5 μg/kg		
		>12 years: 2–3 μg/kg		
		Adults: 1.7 μg/kg		
Growth retardation	rhGH	SC 0.05 mg/kg	QD	Early initiation when growth failure persists after optimal control of feeding, electrolytes and rickets
				Higher doses of phosphate supplementation may be needed
Glucose intolerance	insulin	SC (cfr endocrinology)		Control by blood glucose
				Regular control of Hb A1C
Cysteamine treatment				
Systemic administration	immediate release cysteamine bitartrate (Cystagon®)	1.30–1.95 g/m^2^	QID	Start at low dose (1/6 th of optimal dose), gradual increase over 6–8 weeks
	delayed release cysteamine bitartrate (Procysbi®)	Start with 80 % of the immediate-release form	BID	Gastrointestinal complaints: add proton pomp inhibitors
				Skin lesions (striae, molluscoid tumor at elbows): dose reduction by 25–50 %, control for copper deficiency
				Regular dosing of WBC cystine levels (children x4 per year, adults x1-2 per year)^b^
Corneal cystine crystals	cysteamine eye drops 0.5 %	topical application	8–10 time daily	Yearly eye examination
	cysteamine eye gel (Cystadrops®)		QID	
Varia				
Gastro-intestinal complaints	Proton pump inhibitors omeprazole	<10 kg: 1- mg/kg	BID	
		10–20 kg: 10–20 mg	BID	
		>20 kg: 20–40 mg	BID	

^a^Trough levels of electrolytes and phosphate (before the administration of the next dose) should be measured

^b^Blood for the determination of WBC cystine levels should be taken 6 h after Cystagon® and 12 h after Procysbi® administration

### Therapeutic monitoring

The current gold standard in the therapeutic monitoring of cystinotic patients is the WBC cystine assay. Theoretically, a more specific and ideal therapeutic monitor would be the direct assessment of the fluctuating lysosomal cystine load in different tissues in response to treatment; however, the invasiveness of tissue samples is prohibitive, especially in children. WBCs offer the second best option, and since cystine accumulates preferentially in polymorphonuclear leucocytes but not in lymphocytes, granulocyte separation is preferred to a mixed leucocyte population [119]; however, the large blood volume needed, the analyte instability during transportation, the difficult technique and the unavailability of the assay in many countries, all make the WBC cystine assay far from being perfect as a therapeutic monitor. Furthermore, the extremely short life span of polymorphonuclear leucocytes (≈12 h) might not be ideal for the long term follow-up in patients with unstable compliance [77].

Recently, several non-invasive immunological markers have been proposed to assess the disease activity upon diagnosis and during follow up of cysteamine treatment. The immune system is expected to play a major role in the pathogenesis of nephropathic cystinosis and its rapid progression to ESRD unlike other types of hereditary Fanconi syndromes. Prencipe et al. detected the stimulation of the inflammasome related cytokines: IL-1β, IL-6 and IL-18 in human peripheral mononuclear cells when exposed to cystine crystals, in the plasma of cystinotic patients and in the serum and tissues of *Ctns* knocked-out mice [120]. On the other hand, we reported the significant elevation of the macrophage marker chitotriosidase in cystinotic patients. Moreover, control human macrophages were potently activated in vitro when exposed to different concentrations of cystine crystals through the significant elevations of TNF-α and chitotriosidase in both supernatant and cell lysate. Chitotriosidase activities were also significantly elevated in the plasma of cystinotic knocked-out versus wild-type mice [73]. These immune based markers could be promising indirect indicators of the disease severity and hence the response to treatment, as the cystine crystal accumulation in cystinosis is the main motive behind their release. Besides, they are much more stable and less technically demanding than the WBC cystine assay. Another possible therapeutic monitor of the cystine crystal load needing further evaluation is the in vivo reflectance confocal microscopy of the skin [76].

### Prognosis

Since it was first reported in early twentieth century, prognosis of cystinosis has improved dramatically, particularly with the advent of cystine-depleting treatment and renal replacement therapy (dialysis and kidney transplantation) in the early 1980s. Consequently more patients are increasingly growing into adulthood instead of succumbing to ESRD by late 1st or early 2nd decade of life. With the increased life expectancy more long-term complications are being recognized and reported, that did not have enough time to evolve in the pediatric age group [100].

North American Pediatric Renal Transplant Cooperative Study suggests that the outcome of renal transplantation is favorable in patients with a primary diagnosis of cystinosis [94]. A large European observational registry study reported a significant delay in the age of initiation of renal replacement therapy in nephropathic cystinosis patients (0.15 year per calendar year, 95 % confidence interval: 0.1–0.21 year) which wasn’t observable in a matched cohort of non-cystinotic pediatric patients who started renal replacement therapy in the past 2 decades [101]. In an adult patient cohort, renal transplant recipients with cystinosis had a better long-term outcome than other renal transplant recipients. Authors confirmed, by multivariate analysis, that cystinosis is an independent protective factor for graft survival [96].

Nowadays cystinosis is increasingly being diagnosed at younger age allowing early and adequate initiation of cystine-depleting therapy which significantly prevents, or at least delays, the complications of the disease. That being said, adherence to therapy is critical to improved clinical outcomes. In patients with poor compliance to frequent dosing formulation, the administration of the newly developed delayed-release formulation is likely to improve patient compliance resulting in fewer long-term complications of cystinosis and improved quality of life. Even though cystinosis does not recur in the graft after renal transplantation, yet it continues to progress in other organs and tissues causing complications that may worsen the prognosis, hence the need to continue cysteamine therapy even after kidney transplantation.

### Future prospects and unresolved questions

Recent studies linked cystinosin deficiency in cystinosis to other pathophysiologic mechanisms not related to cystine accumulation such as altered vesicle trafficking and impaired mTOR signaling [121–123], thus a better understanding of the pathogenic mechanisms of cystinosis is highly needed to plan and develop more efficient therapeutic targets. Multiple cell and animal cystinotic models have been developed to better understand and characterize the different phenotypic features of the disease. Immortalized cell lines for cystinotic proximal tubular epithelial cells and podocytes have been established and sustained from either biopsy material or exfoliated cells in urine [122–125]. The mouse model for cystinosis has been also available for over a decade now [126]. Although, it can accumulate cystine in most organs, many important phenotypic features like tubulopathy and renal failure were not expressed in the initial model. A second mouse model was later developed on a pure C57BL/6 background that avoided some of the pitfalls of the first one [127]. These cell and animal models provide indispensable tools to study pathologic and molecular mechanisms of the disease. They can be also used to evaluate the in vitro and in vivo responses to experimental new therapeutic drugs and different therapeutic strategies.

With the current rapid advance in the technology of tandem mass spectrometry, the sensitivity of recent machines are almost two to three orders of magnitude the older ones, thus the development of a suitable method for the newborn screening of cystinosis can be applicable in the near future. An interesting approach is the quantification of the deficient protein cystinosin by peptide Immunoaffinity enriched LC-MS/MS analysis. The technology has already been applied for the detection of signature proteins for three primary immunodeficiency diseases: severe combined immunodeficiency (SCID), Wiskott–Aldrich syndrome (WAS), and X-linked agammaglobulinemia (XLA) [128].

Although, the newly investigated diagnostic markers look promising for the monitoring of disease activity and treatment response [73, 76, 120], longitudinal clinical studies are strongly needed to validate these observations. Laboratory markers as chitotriosidase and interleukins, while being much easier to sample and measure than WBC cystine, are not strictly specific for cystinosis, thus sensitivity and specificity issues need to be handled carefully before determining their utility as therapeutic monitors. On the other hand, the monitoring of the in vivo dermal cystine crystals by confocal microscopy, while being extremely specific for the disease, needs a great deal of experience to operate the instrument and interpret the results. Whether this experience can be available for routine clinical use or not, only the future can tell.

The search for therapeutic substitutes for cysteamine is now strongly ongoing. Adverse effects and compliance issues are still hindering the full capacity of the drug even after the development of the longer acting formulation. The therapeutic focus now is not just cystine depletion, but also how to alleviate other possible harmful pathogenic mechanisms in cystinosis such as inflammation, autophagy and oxidative stress [120, 129, 130]. New promising therapeutics that can target these different disease mechanisms are being currently evaluated in cell and animal models. Hematopoietic stem cell transplantation in humans is another interesting therapeutic option, raising new hopes in finding a cure for cystinosis and further improving long-term clinical outcomes. Being highly successful in the mice model [131, 132], therapeutic human trials are currently being planned but whether it is going to be as successful and safe in humans as in mice, is yet to be determined.

## Conclusions

Cystinosis is a systemic disease that needs a multilevel clinical collaboration to rapidly diagnose and properly treat. The current diagnostic and therapeutic regimens made it possible for the transition of most cystinotic patients to adulthood; however, the search for more efficient screening and better therapeutic options through unravelling the basic pathogenic mechanisms of the disease will surely hold the promise to a better future.

## Consent

Written informed consents were obtained from patients’ parents for the publication of images in this report.

## References

[1] Abderhalden E (1903). Familiare cystindiathese. Z Physiol Chem.

[2] Lignac GOE (1924). Uber storung des cystinstoffwechsels bei kindern. Deutsch Arch Klin Med.

[3] Fanconi G (1931). Die nicht diabetischen glykosurien und hyperglykaemien des aelteren kindes. Jb Kinderheilk.

[4] Nesterova G, Gahl WA (2013). Cystinosis: the evolution of a treatable disease. Pediatr Nephrol.

[5] Thoene JG, Oshima RG, Crawhall JC, Olson DL, Schneider JA (1976). Cystinosis. Intracellular cystine depletion by aminothiols in vitro and in vivo. J Clin Invest.

[6] Liu B, Du H, Rutkowski R, Gartner A, Wang X (2012). LAAT-1 is the lysosomal lysine/arginine transporter that maintains amino acid homeostasis. Science.

[7] Jézégou A, Llinares E, Anne C, Kieffer-Jaquinod C, O’Regan S, Aupetit J, Chabli A, Sagné C, Debacker C, Chadefaux-Vekemans B, Journet A, André B, Gasnier B (2012). Heptahelical protein PQLC2 is a lysosomal cationic amino acid exporter underlying the action of cysteamine in cystinosis therapy. Proc Natl Acad Sci U S A.

[8] Gahl WA, Thoene JG, Schneider JA (2002). Cystinosis. N Engl J Med.

[9] Cochat P, Cordier B, Lacote C, Said M-H, Broyer M (1999). Cystinosis: Epidemiology in France. Cystinosis.

[10] Meikle PJ, Hopwood JJ, Clague AE, Carey WF (1999). Prevalence of lysosomal storage disorders. JAMA.

[11] Manz F, Gretz N (1985). Cystinosis in the Federal Republic of Germany. J Inherit Metab Dis.

[12] Ebbesen F, Mygind KI, Holck F (1976). Infantile nephropathic cystinosis in Denmark. Danish Med Bull.

[13] Hult M, Darin N, von Döbeln U, Månsson JE (2014). Epidemiology of lysosomal storage diseases in Sweden. Acta Paediatr.

[14] Bois E, Feingold J, Frenay P, Briard ML (1976). Infantile cystinosis in France: genetics, incidence, geographic distribution. J Med Genet.

[15] De Braekeleer M (1991). Hereditary disorders in Saguenay-Lac-St-Jean (Quebec, Canada). Hum Hered.

[16] Hutchesson AC, Bundey S, Preece MA, Hall SK, Green A (1998). A comparison of disease and gene frequencies of inborn errors of metabolism among different ethnic groups in the West Midlands. UK J Med Genet.

[17] Elmonem MA, Mahmoud IG, Mehaney DA, Sharaf SA, Hassan SA, Orabi A, Salem F, Girgis MY, El-Badawy A, Abdelwahab M, Salah Z, Soliman NA, Hassan FA, Selim LA (2016). Lysosomal storage disorders in Egyptian children. Ind J Pediatr.

[18] Levtchenko E, van den Heuvel L, Emma F, Antignac C. Clinical utility gene card for: cystinosis. Eur J Hum Genet. 2014;22(5). 10.1038/ejhg.2013.204 PMC3992566 24045844

[19] Shotelersuk V, Larson D, Anikster Y, McDowell G, Lemons R, Bernardini I, Guo J, Thoene J, Gahl WA (1998). CTNS mutations in an American-based population of cystinosis patients. Am J Hum Genet.

[20] Soliman NA, Elmonem MA, van den Heuvel L, Abdel Hamid RH, Gamal M, Bongaers I, Marie S, Levtchenko E (2014). Mutational Spectrum of the CTNS Gene in Egyptian Patients with Nephropathic Cystinosis. JIMD Rep.

[21] Schnaper HW, Cottel J, Merrill S, Marcusson E, Kissane JM, Schackelford GD, So SK, Nelson RD, Cole BR, Smith ML (1992). Early occurence of end-stage renal disease in a patient with infantile cystinosis. J Pediatr.

[22] Long WS, Seashore MR, Siegel NJ, Bia MJ (1990). Idiopathic Fanconi Syndrome with progressive renal failure: a case report and discussion. Yale J Biol Med.

[23] Brodehl J, Hagge W, Gellisen K (1965). Changes in kidney function in cystinosis. I. Inulin, PAH and electrolyete clearance in various stages of the disease. Ann Paediatr.

[24] Baum M (1998). The fanconi syndrome of cystinosis: insights into the pathophysiology. Pediatr Nephrol.

[25] Roth KS, Foreman JW, Segal S (1981). The Fanconi syndrome and mechanisms of tubular transport dysfunction. Kidney Int.

[26] Gaide Chevronnay HP, Janssens V, Van Der Smissen P, N’Kuli F, Nevo N, Guiot Y, Levtchenko E, Marbaix E, Pierreux CE, Cherqui S, Antignac C, Courtoy PJ (2014). Time course of pathogenic and adaptation mechanisms in cystinotic mouse kidneys. J Am Soc Nephrol.

[27] Wilmer MJ, Schoeber JP, van den Heuvel LP, Levtchenko EN (2011). Cystinosis: practical tools for diagnosis and treatment. Pediatr Nephrol.

[28] O’Regan S, Mongeau JG, Robitaille P (1980). A patient with cystinosis presenting with the features of Bartter syndrome. Acta Paediatr Belg.

[29] Ozkan B, Cayir A, Kosan C, Alp H (2011). Cystinosis presenting with findings of barter syndrome. J Clin Res Pediatr Endocrinol.

[30] Gahl WA, Reed GF, Thoene JG, Schulman JD, Rizzo WB, Jonas AJ (1987). Cysteamine therapy for children with nephropathic cystinosis. N Engl J Med.

[31] Besouw M, Levtchenko E (2010). Growth retardation in children with cystinosis. Minerva Pediatr.

[32] Theodoropoulos DS, Shawker TH, Heinrichs C, Gahl WA (1995). Medullary nephrocalcinosis in nephropathiccystinosis. Pediatr Nephrol.

[33] Betend B, Chatelain P, David L, François R (1982). Treatment of rickets caused by infantile cystinosis using 1 alpha-hydroxy vitamin D. Arch Fr Pediatr.

[34] Wilmer MJ, Christensen EI, van den Heuvel LP, Monnens LA, Levtchenko EN (2008). Urinary protein excretion pattern and renal expression of megalin and cubilin in nephropathic cystinosis. Am J Kidney Dis.

[35] Gretz N, Manz F, Augustin R, Barrat TM, Bender-Götze C, Brandis M (1983). Survival time in cystinosis: a collaborative study. Proc Eur Dial Transplant Assoc.

[36] Brodin Sartorius A, Tete MJ, Niaudet P, Antignac C, Guest G, Ottolenghi C, Charbit M, Moyse D, Legendre C, Lesavre P, Cochat P, Servais A (2012). Cysteamine therapy delays the progression of nephropathic cystinosis in late adolescents and adults. Kidney Int.

[37] Middleton R, Bradbury M, Webb N, O’Donoghue D, van’t Hoff W (2003). Cystinosis. A clinico-pathological conference. “from toddlers to twenties and beyond” adult-paediatric nephrology interface meeting, Manchester 2001. Nephrol Dial Transplant.

[38] Servais A, Morinière V, Grünfeld JP, Noël LH, Goujon JM, Chadefaux-Vekemans B, Antignac C (2008). Late-onset nephropathic cystinosis: clinical presentation, outcome, and genotyping. Clin J Am Soc Nephrol.

[39] Anikster Y, Lucero C, Guo J, Huizing M, Shotelersuk V, Bernardini I, McDowell G, Iwata F, Kaiser-Kupfer MI, Jaffe R, Thoene J, Schneider JA, Gahl WA (2000). Ocular non-nephropathic cystinosis: clinical, biochemical, and molecular correlations. Pediatr Res.

[40] Nesterova G, Gahl WA (2008). Nephropathic cystinosis: late complications of a multisystemic disease. Pediatr Nephrol.

[41] Kaiser-Kupfer MI, Caruso RC, Minkler DS, Gahl WA (1986). Long-term ocular manifestations in nephropathic cystinosis. Arch Ophtalmol.

[42] Tsilou ET, Rubin BI, Reed GF, Iwata F, Gahl W, Kaiser-Kupfer MI (2006). Age-related prevalence of anterior segment complications in patients with infantile nephropathic cystinosis. Cornea.

[43] Tsilou ET, Rubin BI, Reed G, Caruso RS, Iwata F, Balog J, Gahl WA, Kaiser-Kupfer MI (2006). Nephropathic cystinosis: posterior segment manifestations and effects of cysteamine therapy. Ophtalmology.

[44] Gahl WA, Kuehl EM, Iwata F, Lindblad A, Kaiser-Kupfer MI (2000). Corneal crystals in nephropathic cystinosis: natural history and treatment with cysteamine eye drops. Mol Genet Metab.

[45] Chan AM, Lynch MJG, Bailey JD, Ezrin C, Fraser D (1970). Hypothyroidism in cystinosis. A clinical, endocrinologic and histologic study involving sixteen patients with cystinosis. Am J Med.

[46] Grünebaum M, Lebowitz RL (1977). Hypothyroidism in cystinosis. Am J Roentgenol.

[47] Gaide Chevronnay HP, Janssens V, Van Der Smissen P, Liao XH, Abid Y, Nevo N, Antignac C, Refetoff S, Cherqui S, Pierreux CE, Courtoy PJ (2015). A mouse model suggests two mechanisms for thyroid alterations in infantile cystinosis: decreased thyroglobulin synthesis due to endoplasmic reticulum stress/unfolded protein response and impaired lysosomal processing. Endocrinology.

[48] Gahl WA, Schneider JA, Thoene JG, Chesney R (1986). The course of nephropathic cystinosis after age 10 years. J Pediatr.

[49] Fivush B, Green OC, Porter CC, Balfe JW, O’Regan S, Gahl WA (1987). Pancreatic endocrine insufficiency in post-transplant cystinosis. Am J Dis Child.

[50] Filler G, Amendt P, von Bredow MA, Rohde W, Ehrich JH (1998). Slowly deteriorating insulin secretion and C-peptide production characterizes diabetes mellitus in infantile cystinosis. Eur J Pediatr.

[51] Fivush B, Flick JA, Gahl WA (1998). Pancreatic exocrine insufficiency in a patient with nephropathic cystinosis. J Pediatr.

[52] Winkler L, Offner G, Krull F, Brodehl J (1993). Growth and pubertal development in nephropathic cystinosis. Eur J Pediatr.

[53] Chik CL, Friedman A, Merriam GR, Gahl WA (1993). Pituitary testicular function in nephropathiccystinosis. Ann Intern Med.

[54] Besouw MT, Kremer JA, Janssen MC, Levtchenko EN (2010). Fertility status in male cystinosis patients treated with cysteamine. Fertil Steril.

[55] Reiss RE, Kuwabara T, Smith ML, Gahl WA (1988). Succesful pregnancy despite placental cystine crystals in a woman with nephropathic cystinosis. N Engl J Med.

[56] Fink JK, Brouwers P, Barton N, Malekzadeh MH, Sato S, Hill S, Cohen WE, Fivush B, Gahl WA (1989). Neurologic complications in long-standing nephropathic cystinosis. Arch Neurol.

[57] Broyer M, Tete MJ, Guest G, Bertheleme JP, Labrousse F, Poisson M (1996). Clinical polymorphism of cystinosis encephalopathy. Results of treatment with cysteamine. J Inherit Metab Dis.

[58] Nichols SL, Press GA, Schneider JA, Trauner DA (1990). Cortical atrophy and cognitive performance in infantile nephropathic cystinosis. Pediatr Neurol.

[59] Trauner DA, Chase C, Scheller J, Katz B, Schneider JA (1988). Neurologic and cognitive deficits in children with cystinosis. J Pediatr.

[60] Dogulu CF, Tsilou E, Rubin B, Fitsgibbon EJ, Kaiser-Kupfer MI, Rennert OM, Gahl WA (2004). Idiopathic Intracranial hypertension in cystinosis. J Pediatr.

[61] Delgado G, Schatz A, Nichols S, Appelbaum M, Trauner D (2004). Behavioral profiles of children with infantile nephropathic cystinosis. Dev Med Child Neurol.

[62] Besouw MT, Hulstijn-Dirkmaat GM, van der Rijken RE, Cornelissen EA, van Dael CM, Vande Walle J, Lilien MR, Levtchenko EN (2010). Neurocognitive functioning in school-aged cystinosis patients. J Inherit Metab Dis.

[63] Bava S, Theilmann RJ, Sach M, May SJ, Frank LR, Hesselink JR, Vu D, Trauner DA (2010). Developmental changes in cerebral white matter microstructure in a disorder of lysosomal storage. Cortex.

[64] Gahl WA, Dalakas MC, Charnas L, Chen KT, Pezeshkpour GH, Kuwabara T (1988). Myopathy and cystine storage in muscles in a patient with nephropathic cystinosis. N Engl J Med.

[65] Vester U, Schubeter M, Offner G, Brodehl J (2000). Distal myopathy in nephropathic cystinosis. Pediatr Nephrol.

[66] Anikster Y, Lacbawan F, Brantly M, Gochuico BL, Avila NA, Travis W, Gahl WA (2001). Pulmonary dysfunction in adults with nephropathic cystinosis. Chest.

[67] Sonies BC, Almajid P, Kleta R, Bernardini I, Gahl WA (2005). Swallowing dysfunction in 101 patients with nephropathic cystinosis – benefit of long-term cysteamine therapy. Medicine.

[68] Guillet G, Sassolas B, Fromentoux S, Gobin E, Leroy JP (1998). Skin storage of cystine and premature skin ageing in cystinosis. Lancet.

[69] Chiaverini C, Sillard L, Flori E, Ito S, Briganti S, Wakamatsu K, Fontas E, Berard E, Cailliez M, Cochat P, Foulard M, Guest G, Niaudet P, Picardo M, Bernard FX, Antignac C, Ortonne JP, Ballotti R (2012). Cystinosin is a melanosomal protein that regulates melanin synthesis. FASEB J.

[70] Oshima RG, Willis RC, Furlong CE, Schneider JA (1974). Binding assays for amino acids. The utilization of a cystine binding protein from Escherichia coli for the determination of acid-soluble cystine in small physiological samples. J Biol Chem.

[71] de Graaf-Hess A, Trijbels F, Blom H (1999). New method for determining cystine in leukocytes and fibroblasts. Clin Chem.

[72] Chabli A, Aupetit J, Raehm M, Ricquier D, Chadefaux-Vekemans B (2007). Measurement of cystine in granulocytes using liquid chromatography-tandem mass spectrometry. Clin Biochem.

[73] Elmonem MA, Makar SH, van den Heuvel L, Abdelaziz H, Abdelrahman SM, Bossuyt X, Janssen MC, Cornelissen EA, Lefeber DJ, Joosten LA, Nabhan MM, Arcolino FO, Hassan FA, Gaide Chevronnay HP, Soliman NA, Levtchenko E (2014). Clinical utility of chitotriosidase enzyme activity in nephropathic cystinosis. Orphanet J Rare Dis.

[74] Taranta A, Wilmer MJ, van den Heuvel LP, Bencivenga P, Bellomo F, Levtchenko EN, Emma F (2010). Analysis of CTNS gene transcripts in nephropathic cystinosis. Pediatr Nephrol.

[75] Soliman NA, El-Baroudy R, Rizk A, Bazaraa H, Younan A (2009). Nephropathic cystinosis in children: An overlooked disease. Saudi J Kidney Dis Transpl.

[76] Chiavérini C, Kang HY, Sillard L, Berard E, Niaudet P, Guest G, Cailliez M, Bahadoran P, Lacour JP, Ballotti R, Ortonne JP (2013). In vivo reflectance confocal microscopy of the skin: a noninvasive means of assessing body cystine accumulation in infantile cystinosis. J Am Acad Dermatol.

[77] Besouw MT, Van Dyck M, Cassiman D, Claes KJ, Levtchenko EN (2015). Management dilemmas in pediatric nephrology: Cystinosis. Pediatr Nephrol.

[78] Wamelink MM, Struys EA, Jansen EE, Blom HJ, Vilboux T, Gahl WA, Kömhoff M, Jakobs C, Levtchenko EN (2011). Elevated concentrations of sedoheptulose in bloodspots of patients with cystinosis caused by the 57-kb deletion: implications for diagnostics and neonatal screening. Mol Genet Metab.

[79] Heil SG, Levtchenko E, Monnens LA, Trijbels FJ, Van der Put NM, Blom HJ (2001). The molecular basis of Dutch infantile nephropathic cystinosis. Nephron.

[80] Kiehntopf M, Varga RE, Koch HG, Beetz C (2012). A homemade MLPA assay detects known CTNS mutations and identifies a novel deletion in a previously unresolved cystinosis family. Gene.

[81] Bendavid C, Kleta R, Long R, Ouspenskaia M, Muenke M, Haddad BR, Gahl WA (2004). FISH diagnosis of the common 57-kb deletion in CTNS causing cystinosis. Hum Genet.

[82] States B, Blazer B, Harris D, Segal S (1975). Prenatal diagnosis of cystinosis. J Pediatr.

[83] Jackson M, Young E (2005). Prenatal diagnosis of cystinosis by quantitative measurement of cystine in chorionic villi and cultured cells. Prenat Diagn.

[84] Emma F, Nesterova G, Langman C, Labbé A, Cherqui S, Goodyer P, Janssen MC, Greco M, Topaloglu R, Elenberg E, Dohil R, Trauner D, Antignac C, Cochat P, Kaskel F, Servais A, Wühl E, Niaudet P, Van’t Hoff W, Gahl W, Levtchenko E (2014). Nephropathic cystinosis: an international consensus document. Nephrol Dial Transplant.

[85] Loirat C. Symptomatic therapy. In: Broyer M, editors. Cystinosis, 1st edn Elsevier; Paris: 1999.pp.97–102.

[86] Cochat P, Pichault V, Bacchetta J, Dubourg L, Sabot JF, Saban C, Daudon M, Liutkus A (2010). Nephrolithiasis related to inborn metabolic diseases. Pediatr Nephrol..

[87] Gahl WA, Bernardini I, Dalakas M, Rizzo WB, Harper GS, Hoeg JM, Hurko O, Bernar J (1988). Oral carnitine therapy in children with cystinosis and renal Fanconi syndrome. J Clin Invest.

[88] Gahl WA, Bernardini IM, Dalakas MC, Markello TC, Krasnewich DM, Charnas LR (1993). Muscle carnitine repletion by long-term carnitine supplementation in nephropathic cystinosis. Pediatr Res.

[89] Elenberg E, Norling LL, Kleinman RE, Ingelfinger JR (1998). Feeding problems in cystinosis. Pediatr Nephrol.

[90] Besouw MT, Van Dyck M, Francois I, Van Hoyweghen E, Levtchenko EN (2012). Detailed studies of growth hormone secretion in cystinosis patients. Pediatr Nephrol.

[91] Greco M, Brugnara M, Zaffanello M, Taranta A, Pastore A, Emma F (2010). Long-term outcome of nephropathic cystinosis: a 20-year single-center experience. Pediatr Nephrol.

[92] Levtchenko E, Blom H, Wilmer M, van den Heuvel L, Monnens L (2003). ACE inhibitor enalapril diminishes albuminuria in patients with cystinosis. Clin Nephrol.

[93] Spear GS, Gubler MC, Habib R, Broyer M (1989). Renal allografts in cystinosis and mesangial demography. Clin Nephrol.

[94] Kashtan CE, McEnery PT, Tejani A, Stablein DM (1995). Renal allograft survival according to primary diagnosis: a report of the North American Pediatric Renal Transplant Cooperative Study. Pediatr Nephrol.

[95] Rigden SP, Broyer M (1999). Data from the ERA-EDTA registry. Cystinosis.

[96] Cohen C, Charbit M, Chadefaux-Vekemans B, Giral M, Garrigue V, Kessler M, Antoine C, Snanoudj R, Niaudet P, Kreis H, Legendre C, Servais A (2015). Excellent long-term outcome of renal transplantation in cystinosis patients. Orphanet J Rare Dis.

[97] Ehrich JH, Brodehl J, Byrd DI, Hossfeld S, Hoyer PF, Leipert KP, Offner G, Wolff G (1991). Renal transplantation in 22 children with nephropathic cystinosis. Pediatr Nephrol.

[98] Schneider JA, Clark KF, Greene AA, Reisch JS, Markello TC, Gahl WA, Thoene JG, Noonan PK, Berry KA (1995). Recent advances in the treatment of cystinosis. J Inherit Metab Dis.

[99] Markello TC, Bernardini IM, Gahl WA (1993). Improved renal function in children with cystinosis treated with cysteamine. N Engl J Med.

[100] Gahl WA, Balog JZ, Kleta R (2007). Nephropathic cystinosis in adults: natural history and effects of oral cysteamine therapy. Ann Intern Med.

[101] Van Stralen KJ, Emma F, Jager KJ, Verrina E, Schaefer F, Laube GF, Lewis MA, Levtchenko EN (2011). Improvement in the renal prognosis in nephropathic cystinosis. Clin J Am Soc Nephrol.

[102] Kleta R, Bernardini I, Ueda M, Varade WS, Phrnphutkul C, Krasnewich D, Gahl WA (2004). Long-term follow-up of well-treated nephropathic cystinosis patients. J Pediatr.

[103] Kimonis VE, Troendle J, Rose SR, Ynag ML, Markello TC, Gahl WA (1995). Effects of early cysteamine therapy on thyroid function and growth in nephropathic cystinosis. J Clin Endocrinol Metab.

[104] Van’t Hoff WG, Gretz N (1995). The treatment of cystinosis with cysteamine and phosphocysteamine in the United Kingdom and Eire. Pediatr Nephrol.

[105] Labbé A, Baudouin C, Deschênes G, Loirat C, Charbit M, Guest G, Niaudet P (2014). A new gel formulation of topical cysteamine for the treatment of corneal cystine crystals in cystinosis: the Cystadrops OCT-1 study. Mol Genet Metab.

[106] Nesterova G, Williams C, Bernardini I, Gahl WA (2015). Cystinosis: renal glomerular and renal tubular function in relation to compliance with cystine-depleting therapy. Pediatr Nephrol.

[107] Wenner WJ, Murphy JL (1997). The effects of cysteamine on the upper gastrointestinal tract of children with cystinosis. Pediatr Nephrol.

[108] Dohil R, Newbury RO, Sellers ZM, Deutsch R, Schneider JA (2003). The evaluation and treatment of gastrointestinal disease in children with cystinosis receiving cysteamine. J Pediatr.

[109] Dohil R, Fidler M, Barshop BA, Gangoiti J, Deutsch R, Martin M, Schneider JA (2006). Understanding intestinal cysteamine bitartrate absorption. J Pediatr.

[110] Dohil R, Fidler M, Barshop B, Newbury R, Sellers Z, Deutsch R, Schneider J (2005). Esomeprazole therapy for gastric acid hypersecretion in children with cystinosis. Pediatr Nephrol.

[111] Besouw M, Blom H, Tangerman A, de Graaf-Hess A, Levtchenko E (2007). The origin of halitosis in cystinotic patients due to cysteamine treatment. Mol Genet Metab.

[112] Ivanova E, De Leo MG, De Matteis MA, Levtchenko E (2014). Cystinosis: clinical presentation, pathogenesis and treatment. Pediatr Endocrinol Rev.

[113] Besouw MT, Bowker R, Dutertre JP, Emma F, Gahl WA, Greco M, Lilien MR, McKiernan J, Nobili F, Schneider JA, Skovby F, van den Heuvel LP, Hoff WG Van’t, Levtchenko EN. Cysteamine toxicity in patients with cystinosis. J Pediatr. 2011;159:1004–11. 10.1016/j.jpeds.2011.05.057 21784456

[114] Corden BJ, Schulman JD, Schneider JA, Thoene JG (1981). Adverse reactions to oral cysteamine use in nephropathic cystinosis. Dev Pharmacol Ther.

[115] Beckman DA, Mullin JJ, Assadi FK (1998). Developmental toxicity of cysteamine in the rat: effects on embryo-fetal development. Teratology.

[116] Assadi FK, Mullin JJ, Beckman DA (1998). Evaluation of the reproductive and developmental safety of cysteamine in the rat: effects on female reproduction and early embryonic development. Teratology.

[117] Levtchenko EN, van Dael CM, de Graaf-Hess AC, Wilmer MJ, van den Heuvel LP, Monnens LA, Blom HJ (2006). Strict cysteamine dose regimen is required to prevent nocturnal cystine accumulation in cystinosis. Pediatr Nephrol.

[118] Dohil R, Fidler M, Gangoiti JA, Kaskel F, Schneider JA, Barshop BA (2010). Twice-daily cysteamine bitartrate therapy for children with cystinosis. J Pediatr.

[119] Levtchenko E, de Graaf-Hess A, Wilmer M, van den Heuvel L, Monnens L, Blom H (2004). Comparison of cystine determination in mixed leukocytes vs polymorphonuclear leukocytes for diagnosis of cystinosis and monitoring of cysteamine therapy. Clin Chem.

[120] Prencipe G, Caiello I, Cherqui S, Whisenant T, Petrini S, Emma F, De Benedetti F (2014). Inflammasome activation by cystine crystals: implications for the pathogenesis of cystinosis. J Am Soc Nephrol.

[121] Andrzejewska Z, Nevo N, Thomas L, Chhuon C, Bailleux A, Chauvet V, Courtoy PJ, Chol M, Guerrera IC, Antignac C. Cystinosin is a Component of the Vacuolar H + −ATPase-Ragulator-Rag Complex Controlling Mammalian Target of Rapamycin Complex 1 Signaling. J Am Soc Nephrol. 2015 [Epub ahead of print]. 10.1681/ASN.2014090937 PMC4884097 26449607

[122] Ivanova EA, De Leo MG, Van Den Heuvel L, Pastore A, Dijkman H, De Matteis MA, Levtchenko EN (2015). Endo-lysosomal dysfunction in human proximal tubular epithelial cells deficient for lysosomal cystine transporter cystinosin. PLoS One.

[123] 123- Ivanova EA, van den Heuvel LP, Elmonem MA, De Smedt H, Missiaen L, Pastore A, Mekahli D, Bultynck G, Levtchenko E. Altered mTOR signalling in nephropathic cystinosis. J Inherit Metab Dis. 2016 [In press]. 10.1007/s10545-016-9919-z 26909499

[124] Jansen J, Schophuizen CMS, Wilmer MJ, Lahham SH, Mutsaers HA, Wetzels JF, Bank RA, van den Heuvel LP, Hoenderop JG, Masereeuw R (2014). A morphological and functional comparison of proximal tubule cell lines established from human urine and kidney tissue. Exp Cell Res.

[125] Oliveira Arcolino F, Tort Piella A, Papadimitriou E, Bussolati B, Antonie DJ, Murray P, van den Heuvel L, Levtchenko E (2015). Human Urine as a Noninvasive Source of Kidney Cells. Stem Cells Int.

[126] Cherqui S, Kalatzis V, Forestier L, Poras I, Antignac C (2000). Identification and characterisation of the murine homologue of the gene responsible for cystinosis. Ctns BMC Genomics.

[127] Nevo N, Chol M, Bailleux A, Kalatzis V, Morisset L, Devuyst O, Gubler MC, Antignac C (2010). Renal phenotype of the cystinosis mouse model is dependent upon genetic background. Nephrol Dial Transplant.

[128] Kerfoot SA, Jung S, Golob K, Torgerson TR, Hahn SH (2012). Tryptic peptide screening for primary immunodeficiency disease by LC/MS-MS. Proteomics Clin Appl.

[129] Sansanwal P, Sarwal MM (2010). Abnormal mitochondrial autophagy in nephropathic cystinosis. Autophagy.

[130] Galarreta CI, Forbes MS, Thornhill BA, Antignac C, Gubler MC, Nevo N, Murphy MP, Chevalier RL (2015). The swan-neck lesion: proximal tubular adaptation to oxidative stress in nephropathic cystinosis. Am J Physiol Renal Physiol.

[131] Syres K, Harrison F, Tadlock M, Jester JV, Simpson J, Roy S, Salomon DR, Cherqui S (2009). Successful treatment of the murine model of cystinosis using bone marrow cell transplantation. Blood.

[132] Harrison F, Yeagy BA, Rocca CJ, Kohn DB, Salomon DR, Cherqui S (2013). Hematopoietic stem cell gene therapy for the multisystemic lysosomal storage disorder cystinosis. Mol Ther.

